# Quantitative assessment of intertarget position variations based on 4D-CT and 4D-CBCT simulations in single-isocenter multitarget lung stereotactic body radiation therapy

**DOI:** 10.1007/s00432-024-05836-w

**Published:** 2024-07-24

**Authors:** Siyu Zhang, Chang Guo, Jun Xu, Pudong Qian, Jiali Guo, Tingting Liu, Yifan Wu, Jun Hong, Qi Wang, Xia He, Li Sun

**Affiliations:** 1 Department of Radiotherapy, Jiangsu Cancer Hospital, Jiangsu Institute of Cancer Research, the Affiliated Cancer Hospital of Nanjing Medical University, Nanjing, 210009 China; 2 Department of Radiation Oncology, Dantu People’s Hospital, Anhui, Maanshan, 243100 China; 3 Department of Radiation Oncology, the Affiliated Huaian No. 1 People’s Hospital of Nanjing Medical University, Huaian, 223300 Jiangsu China

**Keywords:** Intertarget position variation, Multiple targets, Lung SBRT, 4D-CBCT

## Abstract

**Background:**

In single-isocenter multitarget stereotactic body radiotherapy (SBRT), geometric miss risks arise from uncertainties in intertarget position. However, its assessment is inadequate, and may be interfered by the reconstructed tumor position errors (RPEs) during simulated CT and cone beam CT (CBCT) acquisition. This study aimed to quantify intertarget position variations and assess factors influencing it.

**Methods:**

We analyzed data from 14 patients with 100 tumor pairs treated with single-isocenter SBRT. Intertarget position variation was measured using 4D-CT simulation to assess the intertarget position variations (Δ*D*) during routine treatment process. Additionally, a homologous 4D-CBCT simulation provided RPE-free comparison to determine the impact of RPEs, and isolating purely tumor motion induced Δ*D* to evaluate potential contributing factors.

**Results:**

The median Δ*D* was 4.3 mm (4D-CT) and 3.4 mm (4D-CBCT). Variations exceeding 5 mm and 10 mm were observed in 31.1% and 5.5% (4D-CT) and 20.4% and 3.4% (4D-CBCT) of fractions, respectively. RPEs necessitated an additional 1–2 mm safety margin. Intertarget distance and breathing amplitude variability showed weak correlations with variation (*R*_s_ = 0.33 and 0.31). The Δ*D* differed significantly by locations (upper vs. lower lobe and right vs. Left lung). Notably, left lung tumor pairs exhibited the highest risk.

**Conclusions:**

This study provide a reliable way to assess intertarget position variation by using both 4D-CT and 4D-CBCT simulation. Consequently, single-isocenter SBRT for multiple lung tumors carries high risk of geometric miss. Tumor motion and RPE constitute a substantial portion of intertarget position variation, requiring correspondent strategies to minimize the intertarget uncertainties.

**Supplementary Information:**

The online version contains supplementary material available at 10.1007/s00432-024-05836-w.

## Background

SBRT is the standard-of-care for early-stage Non-Small Cell Lung Cancer (NSCLC), but recent reports suggest it may also benefit patients with limited or multiple metastases by prolonging survival compared to maintenance chemotherapy (Iyengar et al. 2018; Gomez et al. 2016). Single-isocenter multitarget SBRT clearly has advantage in terms of delivery efficiency, and its dosimetric parameters are not inferior to multi-isocenter treatments (Pokhrel et al. 2019; Sanford et al. 2019). However, multiple targets exhibit two types of motion patterns: a grouped movement synchronized with the lung and an independent movement for each tumor. The former can be properly aligned (Pokhrel et al. 2023); the latter may lead to intertarget position variation, potentially making it challenging to align all the tumors on a single CBCT imaging. This can result in geometric miss and the need of repeated treatment processes (e.g., replacement of the isocenter, replanning, etc.). Thus, accurate estimation of the intertarget position variation is crucial for minimizing geometric misses in single single-isocenter multitarget SBRT.

To date, respiratory motion-induced tumor position variations have been investigated in single-target stereotactic body radiotherapy (SBRT) studies (Sonke et al. 2009; Sharma et al. 2022), but intertarget position variations have rarely been reported (Van Timmeren et al. 2021). Moreover, these studies have focused mainly on interfractional tumor position variation which is the displacement of the time-averaged tumor position on each CBCT images relative to the CT simulated position, while the reconstructed position error (RPE) from the two different image acquisition methods is commonly ignored. The 4D-CT relies on a fan beam rotating around the patient to generate one dimensional projections (slices), capturing moving target only on a few slices over the scanning time (Ford et al. 2003); while 4D-CBCT uses a cone beam source to generate two dimensional projections (planes), capturing moving target on every planes (Sonke et al. 2005). This difference in projections (slices vs. planes) can lead to tumor position errors in reconstructed 4D images. Previous studies have demonstrated that the RPEs may increase to 6 mm between the two image acquisition methods (Clements et al. 2013; Li et al. 2018). This level of error suggests that the omission of RPE consideration could have a material impact on the accuracy of estimating tumor position variations, especially in cases with multiple targets. Any additional uncertainties in intertarget positions may further elevate the risk of geometric misses.

This study quantified the interfractional intertarget position variation by using 4D-CT simulation to assess the geometric uncertainties in routine treatment process of multiple lung targets SBRT. Subsequently, a 4D-CBCT simulation isolated pure motion induced intertarget position variation by homologous imaging method. The resulting RPE-free intertarget position variation was further analyzed based on potential contributing factors, including intertarget distance, amplitude variability and location.

## Methods

### Patients

From February 2021 to August 2023, a total of 14 patients with 69 lung metastases were treated with SBRT in our department. Of these, 59 lesions met the inclusion criteria. These lesions were clearly identifiable within the field of view (FOV) of the CBCT scan using X-ray volume imaging (XVI) version 5.0.3 (Elekta, Stockholm, Sweden). This resulted in a total of 100 tumor pairs for analysis. Details on the patients and tumors/tumor pairs are presented in Table 1. Patients underwent free-breathing SBRT (without coaching) using the BodyFix^®^ double-vacuum immobilization system (Elekta, Stockholm, Sweden).

**Table 1:** Patients and tumors characteristics

	*n* [range]
Total number of patients	14
Non-small cell lung cancer	11
Metastatic cancer	3
Tumor in bilateral lungs	10
Tumor in one lateral lung	4
Number of tumors treated	69
On average	4.9 [2–7]
Number of tumors included	59
On average	4.2 [2–7]
Tumor size (cm^3^)	4.6 [0.3–38.3]
Peak-to-peak amplitude	
5 mm ≤	22
> 5 mm, < 10 mm	15
≥ 10 mm	22
Distance to isocenter (cm)	8.53 [2.6–13.2]
Tumor Locations	
Upper lobe lung	21
Middle lobe lung	5
Lower lobe lung	33
Right lung	34
Left lung	25
Tumor pairs	
Combination 1	
Upper lobe	12
Lower lobe	37
Upper-lower lobe	51
Combination 2	
Right lung	36
Left Lung	16
Bilateral lungs	48

### 4D-CT scan and treatment plan

A multislice CT scanner (40-slice Somatom-Sensation; Siemens, Forchheim, Germany) was used for 4D-CT scanning at a thickness of 1.5 mm. The CT sequences were binned in 10 phases based on the respiratory signal acquired with the RPM system (Varian Medical Systems, Palo Alto). The reconstructed images of all 10 phases were subsequently sent to the Monaco treatment planning system (TPS) (Elekta, Stockholm, Sweden) version 5.11, where the averaged intensity projection (AIP) image was generated and subsequently used as a reference CT for the patient setups. The average position of pairwise tumors on the AIP image referred to the initial intertarget position. The targets were initially delineated on the maximum intensity projection (MIP) image to determine the internal target volume (ITV), which was then copied to the AIP CT. An isotopic 5 mm margin was added to obtain the planning target volume (PTV). The PTV was prescribed with a hypo-fractionated treatment regimen (56 Gy in 7 fractions or 50 Gy in 5 fractions). Volumetric modulated arc therapy (VMAT) plan consisting of 1–2 partial/full co-planar arcs with 6MV-FFF beam were optimized in Monaco TPS with Monte Carlo algorithm.

### Preliminary plan and 4D-CBCT simulation

In the preparation of 4D-CBCT simulated imaging, a preliminary plan was designed. The irradiated isocenter was placed at the center of all target volumes to cover the targets in the FOV of the scanner as much as poosible. The open-field plan (0°, MU 1 or 2) was then created and transmitted to an XVI image acquisition system (Elekta, Stockholm, Sweden) version 5.0.3.

All Patients’ position was re-settled from CT simulation isocenter to irradiated isocenter according to preliminary plan. The 4D-CBCT images were acquired by XVI system integrated in the VersaHD linear accelerator (Elekta, Stockholm, Sweden) with the following acquisition parameters: 120 kV, M20 KV Collimator and an F0 image filter (resulting in a FOV of 276.7 mm × 276.7 mm) at a gantry speed of 90°/min. A clockwise rotation from 180°to 180° was accomplished in 4 min, and 1500 frames were generated. The acquired projections were sorted according to the breathing signal extracted from the diaphragm movement and then reconstructed into 4D dynamic images (Sonke et al. 2005). At the end of reconstruction, a time-weighted average image was generated from the reconstructed 4D images. Dual registration (with a clipbox + mask) was performed to correct patient-setup and tumor motion errors (Sonke et al. 2009). First, a 3D rectangle area (clipbox) including vertebrae, lung and thoracic wall of the affected area was defined, enabling the automatic bony anatomy alignment, where gray-value translation algorithm is used. The followed tumor motion registration uses the gray-value 4D algorithm where a region of interest expanded 5 mm from PTVs (mask) on each of the phases of the 4D images were registered against the planning CT image (AIP image from 4D-CT). The time-weighted average image was transmitted to the Monaco TPS, serving as the baseline in the RPE-free inter-target position error assessment. The imaging and registration method in 4D-CBCT simulation was also adopted in the subsequent daily pretreatment online correction. The registration results were reviewed by a qualified physician to ensure the full coverage of moving targets by PTV as much as possible.

### Intertarget position variation

Intertarget position variation (Δ*D*) refers to the relative position displacement between pairwised targets, that was measured by the Euclidian distance variation in the pairwise tumor centroid.

For each direction the intertarget position variation was given by:

1 $${\Delta D}_{(\text{x},\text{ y },\text{ z})}= {{(D}_{\text{i}}-{D}_{0})}_{(\text{x},\text{ y },\text{ z})}$$

The 3D intertarget position variation was calculated by:

2 $${\Delta D}_{3\text{D }}= \sqrt{{({D}_{xi}-{D}_{x0})}^{2}+{({D}_{yi}-{D}_{y0})}^{2}+{({D}_{zi}-{D}_{z0})}^{2}}$$

where *D* represents the intertarget distance. The subscript 0 refers to the time point at simulation and the subscript i refers to treatment fraction i. The subscript x, y, and z are the tumor centroid coordinates in the left–right (LR), superior-inferior (SI), and anterior–posterior (AP) directions, respectively.

More details in measurements of intertarget position variation was illustrated in form of the chart (supplementary Fig. A.1). If A and B are the pairwised targets, then the distance between them in each direction would be the absolute value of the numerical difference of their coordinates:

3 $${D(A,B)}_{(\text{x},\text{ y },\text{ z})}= |{A}_{(\text{x},\text{ y },\text{ z})}-{B}_{(\text{x},\text{ y },\text{ z})}|$$

The coordinates of the tumor centroid were retrieved from the in-house fusion between the reference image (AIP image from 4D-CT simulation or time-averaged image from 4D-CBCT simulation) and the daily time-averaged CBCT image in each fraction on the Monaco TPS. The transitional and rotational errors of the grouped tumor position error, if not properly aligned during the online correction, were adjusted manually.An optimal alignment would be attained when the interfractional tumor position variation, whether grouped or independent, is minimized. The measured value which passed the 5 mm and 10 mm tolerance were recorded to evaluate the risk of geometric miss. Those resulted from 4D-CBCT simulation were served as RPE-free reference dataset in subgroup assessments and correlation tests. The tumor pairs were assigned to two types of combinations: combination 1 included the subgroups of the tumor pairs in the upper lobe, the upper-lower lobe and the lower lobe; combination 2 included subgroups of the tumor pairs in the ipsilateral (right and left) lung and bilateral lungs. Due to a limited sample size of right middle lobe tumors (*n* = 5), these were analyzed together with right lower lobe tumors. While some studies suggest similar motion patterns between middle and lower lobe tumors (Yoshinori and Hidetoshi 2022), this approach acknowledges the anatomical distinction and avoids potential biases from a small subgroup.

The group mean (GM) and the standard deviation (SD) of the GM (systematic error, ∑) and the root-mean-square error (RMSE) of the SD (random error, σ) were calculated. The safe margin compensating for the intertarget position variation was calculated based on the van Herk formula (Van Herk 2004):

4 $${M}_{95\text{\%}}=2.5\Sigma +1.64\sqrt{{\sigma }^{2}+{\sigma }_{\text{p}}^{2}}-1.64\sigma$$

where *σ*_p_ is the penumbra width modelled by a cumulative Gaussian distribution. For lung tumors, *σ*_p_ was set at 3.2 (Van Herk et al. 2000). The equation related the margin from the GTV to the PTV necessary to deliver at least 95% of the prescribed dose to the clinical target volume for 90% of the population.

The association between RPE-free intertarget position variations and intertarget distances was evaluated. Moreover, to investigate whether breathing amplitude variability affects the intertarget position variation, the initial intertarget amplitude difference at simulation was used as baseline to calculate the variability:

5 $$\Delta {(M}_{A}-{M}_{B})={({M}_{A}-{M}_{B})}_{i}-{({M}_{A}-{M}_{B})}_{0}$$

where *M* denotes the peak-to-peak amplitude (magnitude of tumor motion).The inter-target amplitude difference was calculated by subtracting the amplitudes of target A and B. Breathing amplitude variability $\Delta {(M}_{A}-{M}_{B})$ was then determined by subtracting the inter-target amplitude difference at the simulation time point (time point 0) from the inter-target amplitude difference at treatment fraction i.

### Statistical analysis

Statistical outliers were defined using a box plot, where an extreme value was defined as a value greater than the interquartile range multiplied by a factor of 3. The intertarget position variation between the 4D-CT and 4D-CBCT groups was tested by the Wilcoxon rank test. The comparison between the RPE-free subgroups was performed by the Kruskal–Wallis test with the Mann‒Whitney test. Correlation analysis was performed using Spearman’s nonparametric regression. Intergroup and intragroup comparisons of geometric miss risk were performed by the χ^2^ test and Bonferroni correction. All the statistical analyses were performed using the IBM^®^ SPSS^®^ V25.0 software package (New York, USA).

## Results

For all the 14 patients, a total of 605 fractions were treated. All the patients tolerated well with single-isocenter multitarget SBRT. Uncorrectable tumor position errors were inspected in 2 patients (21.4%) during their patient-setups, thus the treatment procedure had to repeat (including re-delineation and re-planning).

**Fig. 1:**
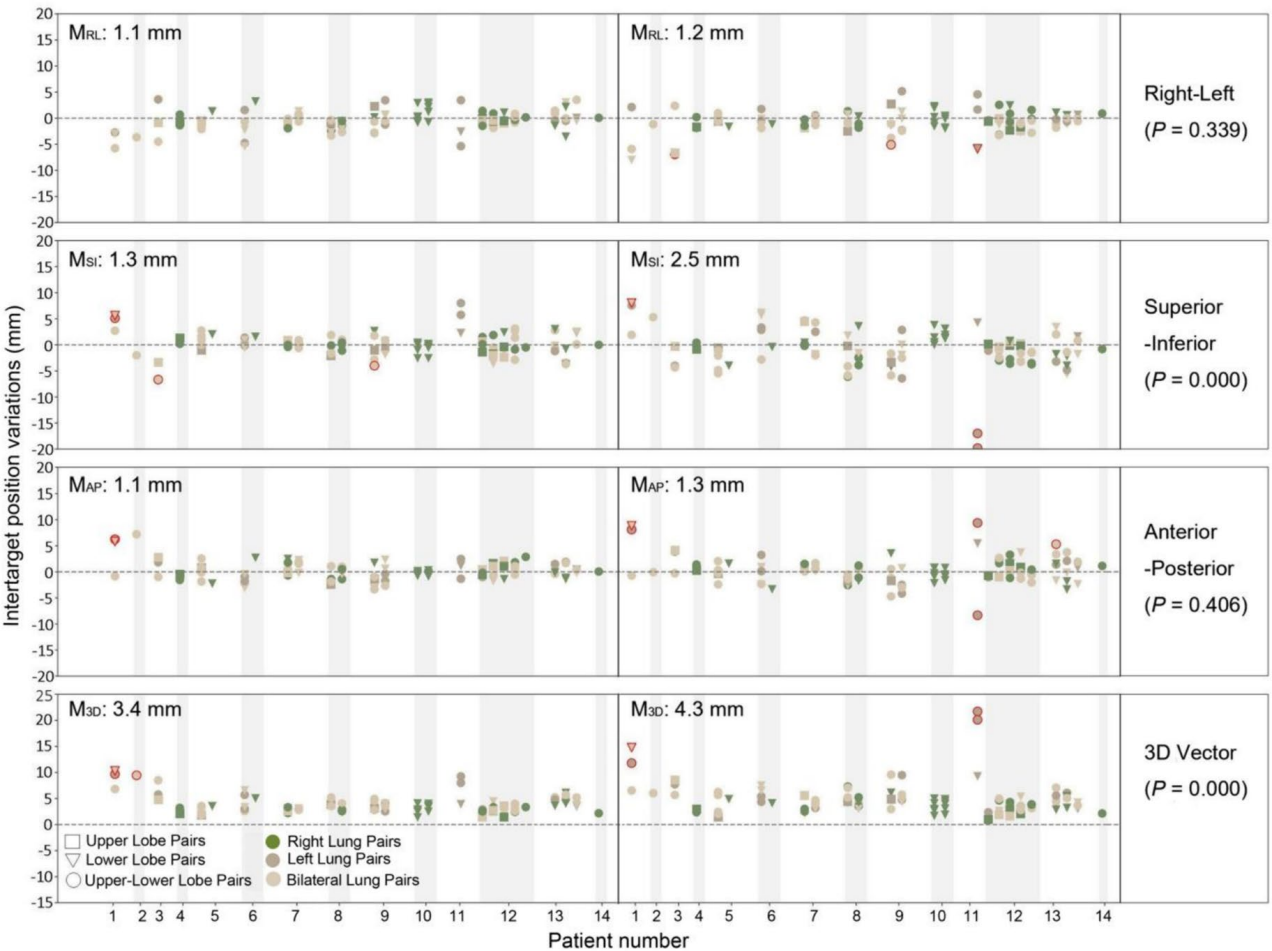
Intertarget position variations in 4D-CT (right) and 4D-CBCT (left) simulation groups for all 14 patients. The negative and positive values referred to the pairwise tumor moving towards or against each other. The outliers were marked with a red outline. M_RL_ denotes the median variations in right-left direction, so forth for the other directions

Figure 1 shows the Δ*D* value in the right-left (RL), superior-inferior (SI), anterior–posterior (AP) direction and in 3D vectors for each tumor pairs (*n* = 100) in both 4D-CT and 4D-CBCT groups. Outliers were detected in 7 and 5 tumor pairs in the 4D-CT and 4D-CBCT groups, respectively. The outliers were all tumor pairs in the bilateral or left lung and were eliminated in the further comparisons of the intertarget position variation and margin calculation but not in the linear regression analysis.

The median Δ*D*_*3D*_ of the 4D-CT group was 4.3 mm, ranging from 1.0 mm to 21.7 mm, and that of the 4D-CBCT group was 3.4 mm, ranging from 1.4 mm to 10.3 mm (with outliers). Among the three orthogonal directions, the absolute average Δ*D* in the SI direction ($\overline{{|\Delta D}_{SI}|}$) in the 4D-CT group was significantly greater than that in the $\overline{{|\Delta D}_{RL}|}$ and $\overline{{|\Delta D}_{AP}|}$ (*P* = 0.000) groups; however, the $\overline{|\Delta D|}$ values in all three directions were comparable (*P* > 0.05) to those in the 4D-CBCT group. The 4D-CBCT group exhibited lower $\overline{{|\Delta D}_{SI}|}$ (*P* = 0.000) and $\overline{{\Delta D}_{3D}}$ (*P* = 0.000) than did the 4D-CT group, which indicated that the significant larger $\overline{{|\Delta D}_{SI}|}$ in the 4D-CT group may probably caused by RPE. Table 2 summarizes the GM, the SD of systematic errors (∑), the RMSE of the SDs of the random errors (σ) of the interfractional intertarget position variations, and the estimated margin in the two groups. Our estimated margin implied that an additional 1–2 mm margin in the SI or AP directions was required to accommodate the RPEs in routine 4D-CT simulation based single-isocenter multitarget SBRT treatment.

**Table 2:** Results of interfractional intertarget position variation and the estimated margin in 4D-CT and 4D-CBCT groups

	4D-CT			4D-CBCT		
	RL (mm)	SI (mm)	AP (mm)	RL (mm)	SI (mm)	AP (mm)
GM	−0.8	−0.7	0.2	−0.4	−0.1	0.2
∑	2.0	3.0	2.3	2.0	2.1	1.7
σ	1.4	2.1	1.8	1.4	2.0	1.9
Margin	7.8	10.6	9.1	7.8	8.8	8.3

*RL* right-lef, *SI* superior-interior, *AP* anterior–posterior, *GM* group mean, *∑* systematic errors, *σ* random errors

**Fig. 2:**
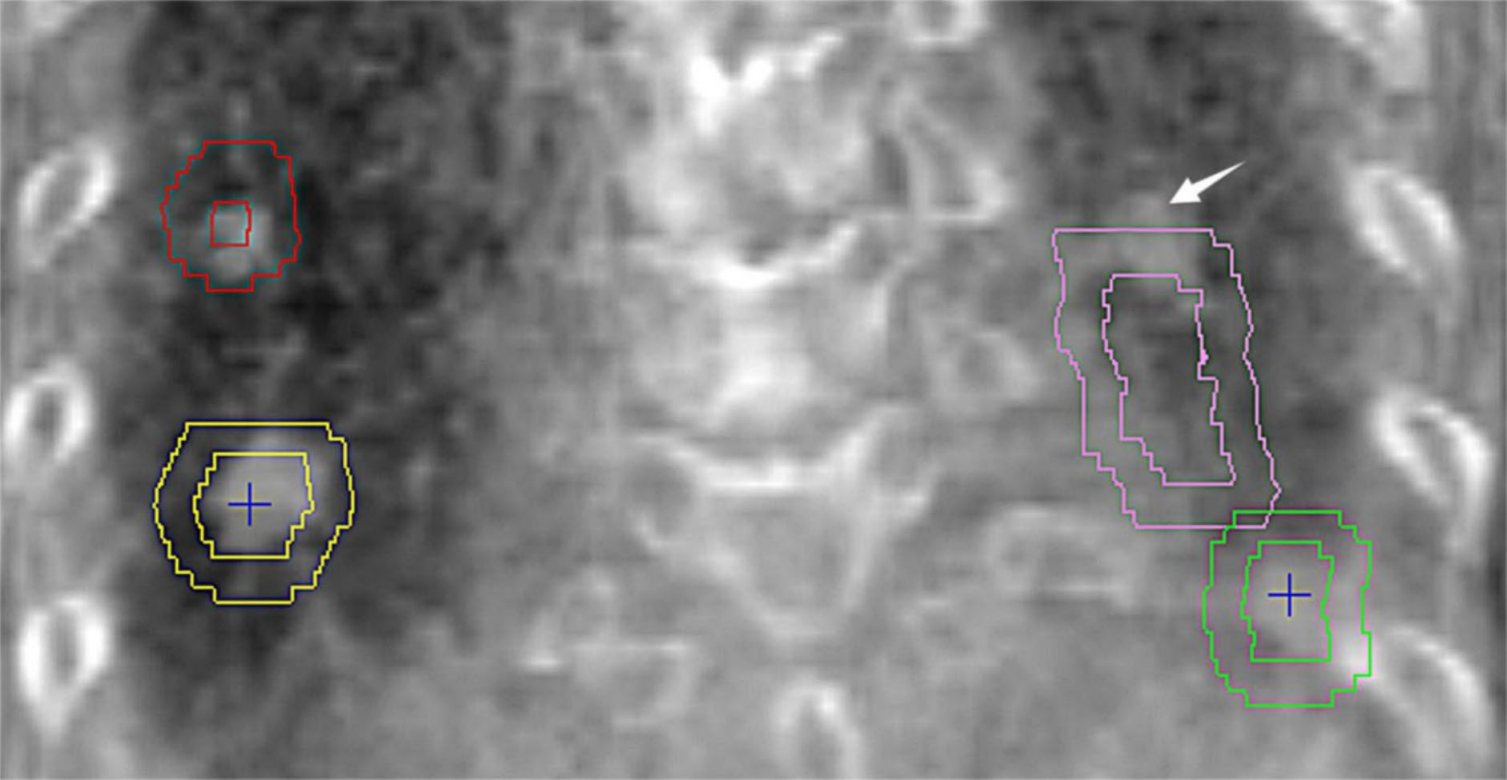
Example of a geometric miss in a patient with multiple tumors in the lower lobe. On the time-averaged CBCT image, 2 out of the 4 tumors reached the border of the PTV (red and yellow outer outlines) as contoured on the reference CT (AIP image), hindering the correction of tumor position, such that one of the tumors (arrow) exceeded the PTV

Among all treated fractions, 5.5% and 3.4% of the ${\Delta D}_{3D}$ exceeded 10 mm tolerance in 4D-CT and 4D-CBCT group, respectively, with *P* value equaled to 0.010. Given that a 5 mm margin is widely used in current clinical practice, these were the fractions at high risk of geometric misses, neglecting the variance in interfractional tumor shape (Sonke et al. 2008). Accordingly, the percentage of treated fractions with an ${\Delta D}_{3D}$ greater than 5 mm were 31.1% and 20.4% in 4D-CT and 4D-CBCT group (*P* = 0.000), respectively. In a group of tumors with more than 2 tumors, if partial tumors reaches their tolerated position, it would be highly possible that the remaining tumors with an intertarget position variation of 5–10 mm would exceed the 5 mm margin of the PTV (Fig. 2). Thus, ${\Delta D}_{3D}$ > 5 mm in a treated fraction is also at risk of geometric misses.

The Spearman rank correlation coefficient (R_s_) between the RPE-free Δ*D*_3D_ and intertarget distance was 0.33 (*P* < 0.001), indicating distance impact. The independent contribution of each orthogonal direction $\Delta {(M}_{A}-{M}_{B})$ to the RPE-free intertarget position variation was all limited (R_s_ = -0.01, 0.05 and 0.02 for the RL, SI and AP directions, respectively); however, for the 3D vectors, a positive correlation (R_s_ = 0.31, *P* = 0.002) between the RPE-free $\overline{{\Delta D}_{3D}}$ and $\Delta {(M}_{A}-{M}_{B})$ was found. The relationships were plotted in supplementary Fig. A.2.

**Fig. 3:**
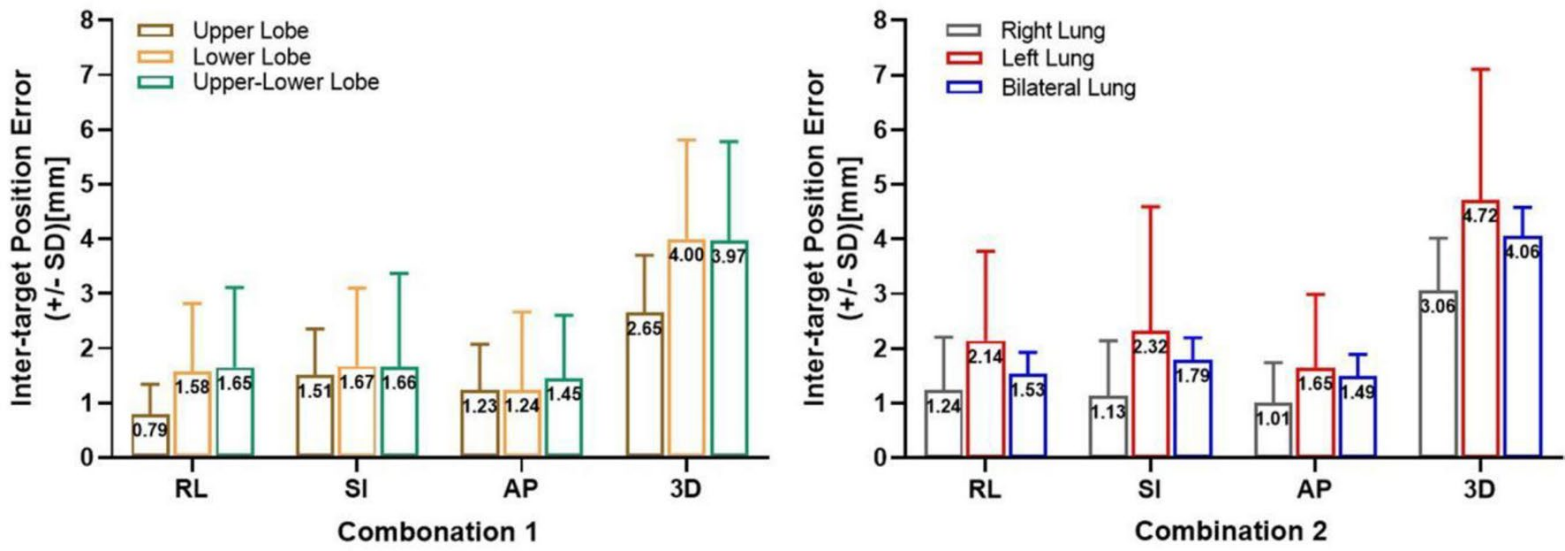
Comparison of the intertarget position variations between subgroups for both combinations

Positional differences were also observed in RPE-free Δ*D.* As shown in Fig. 3, the $\overline{{|\Delta D}_{RL}|}$ and $\overline{{\Delta D}_{3D}}$ of the tumor pairs in the upper lobe were significantly smaller than those of the tumor pairs with at least one tumor in the lower lobe (*P* < 0.05). None of the $\overline{\Delta D}$ values in the three orthogonal directions or in the 3D vector were significantly different between the ipsilateral and bilateral tumor pairs (*P* > 0.05). However, the tumor pairs in the right lung had significantly smaller $\overline{{|\Delta D}_{SI}|}$ and $\overline{{\Delta D}_{3D}}$ than did those in the left lung (*P* < 0.05) and bilateral lung (*P* < 0.05). The $\overline{\Delta D}$ in the left lung was as large as that in the bilateral lungs (*P* > 0.05). The intertarget position variations in terms of the group mean (GM), systematic errors (∑), random errors (σ) and safe margin calculated for both combinations are listed in Table 3.

**Table 3:** Results of intertarget position variations and the estimated margin in subgroups of both combinations

		RL (mm)	SI (mm)	AP (mm)
Combination 1				
Upper Lobe (*n* = 12)	GM	−0.4	−1.0	0.4
	∑	0.9	1.4	1.5
	σ	1.0	1.2	1.2
	Margin	6.1	7.2	7.4
Lower Lobe (*n* = 37)	GM	−0.1	−0.1	0.0
	∑	2.0	1.9	1.7
	σ	1.5	2.4	2.0
	Margin	7.9	8.9	8.2
Upper-lower Lobe (*n* = 51)	GM	−0.8	0.0	0.2
	∑	2.1	2.2	1.9
	σ	1.3	2.1	2.1
	Margin	7.9	9.1	8.6
Combination 2				
Right Lung (*n* = 36)	GM	−0.7	−0.0	0.7
	∑	1.1	1.2	2.1
	σ	1.3	2.1	1.6
	Margin	6.5	7.5	8.6
Left Lung (*n* = 16)	GM	−0.3	0.4	0.5
	∑	2.7	2.5	2.1
	σ	1.6	2.2	2.1
	Margin	9.2	9.8	9.0
Bilateral lung (*n* = 48)	GM	−0.9	−0.3	0.2
	∑	1.9	2.2	2.0
	σ	1.3	2.3	2.1
	Margin	7.6	9.2	8.8

*RL* right-left, *SI* superior-interior, *AP* anterior–posterior, *GM* group mean, *∑* systematic errors; σ random errors

The percentage of treated fractions with a Δ*D*_3D_ greater than 10 mm were low across all six subgroups, which were 0% in upper lobe group, 2.2% in lower lobe group, 3.0% in upper—lower lobe group, 0.9% in right lung group, 5.2% in left lung group and 2.6% in bilateral lung group, respectively. Similarly, the corresponding percentages of treated fractions with a greater than 5 mm were 6.2%, 22.5%, 26.9%, 11.2%, 32.3%, and 29.2% for the upper lobe, lower lobe, upper-lower lobe, right lung, left lung, and bilateral lung groups, respectively. Among fractions with Δ*D*_3D_ exceeding 10 mm, a statistically significant difference was only observed in combination 2 (right vs. left lung): 0.89% compared to 5.21% (*P* = 0.016). Tumor pairs in the left lung almost doubled the risks of geometric miss compared to all other combinations. For fractions exceeding 5 mm Δ*D*_3D_, the statistical differences between subgroups mirrored the trends observed for intertarget position variations across both combinations.

## Discussion

Ensuring accurate tumor positioning is essential for successful lung SBRT treatment. 4D scanning offers a significant advantage over 3D scanning by visualizing tumor motion and defining the tumor center position more precisely. Since target movement speed varies throughout the breathing cycle, the average tumor position generated from 4D scanning is time-weighted. Conversely, the tumor position on static 3D images represents only the geometric mean of the peak-to-peak amplitude, potentially leading to a displacement of a few millimeters from the time-weighted mean position (Li et al. 2012). Several studies have quantified tumor position variation by measuring displacements between 4D-CT simulation and daily verification 4D-CBCT scans (Sonke et al. 2008, 2009). These studies have shown that interfractional tumor position variation can range from a few submillimeters to over 15 mm, depending on the tumor motion. Therefore, it is reasonable to believe that intertarget position variation could be substantial. In this study, we observed a median intertarget position variation of 4.3 mm, with large variations exceeding 5 mm and 10 mm occurring in 5%-31% of fractions. This suggests that treating multiple lung targets with a single isocenter approach may carry a high risk in current clinical practice. In contrast, Van Timmeren et al. (2021) reported generally small intertarget position variations, averaging approximately 1–2 mm. This discrepancy might be attributed to their use of 3D imaging (planning CT and pretreatment CBCT), which may introduce respiratory blurring of the target, potentially affecting registration accuracy. Similar studies are scarce. Further research with larger sample sizes and additional clinical data is necessary for definitive validation.

Driven by diaphragm motion, tumor motion is predominant in SI direction (Bissonnette et al. 2009). Consequently, most reported interfractional tumor position variations also occur primarily in the SI direction (Sonke et al. 2008; Jin et al. 2015). However, a study by Li et al. (2021) using 4D-CBCT simulation for assessment found less pronounced SI direction variation compared to previous studies. They attributed this finding to the use of homologous image registration between the 4D-CBCT simulation and daily 4D-CBCT scans. We observed a similar trend in intertarget position variation using 4D-CBCT simulation, suggesting a potential influence of RPEs. Steiner et al. (2019) compared implant-based reconstructed tumor motion, considered the gold standard, with that of 4D-CT and 4D-CBCT images. Their findings showed that implant-based motion was more consistent with 4D-CBCT reconstruction, particularly in the SI direction. This suggests that 4D-CT simulation may significantly underestimate SI direction tumor motion. The underestimation in 4D-CT is likely caused by the lower sampling rate of breathing cycles (typically 7–11 cycles) during the scan (Ford et al. 2003). Previous phantom studies suggest that RPEs may be less prominent during smooth, sinusoidal breathing patterns (Wang et al. 2013). However, irregular breathing patterns can significantly shift tumor centroid positions (Clements et al. 2013). Therefore, maintaining a regular breathing pattern during treatment may help minimize the impact of RPEs.

We investigated contributing factors underlying intertarget position variation based on purely motion induced intertarget position variation. The weak correlation coefficient suggested that intertarget distance and breathing amplitude variability may play a role to intertarget position variation, but the influence is not straightforward. Theoretically, as distance increases, they should become more sensitive to slight anatomic deformation during patient setups, and may exhibit greater unsynchronized motion. Further, tumor motion amplitude was previously considered a key factor in tumor position variation, but the scenario is complex even for single targets. Studies reported linear relationships between interfractional variation and amplitude ranging from 0.47–0.58 during breathing cycle (Sonke et al. 2008). Liang et al. (2018) found a stronger correlation in the SI direction (R = 0.699) compared to the AP direction (R = 0.329) using logfile-based motion data. This correlation was even weaker eliminating the factor of RPEs, where correlation coefficients was only 0.353 and -0.227 for SI and AP directions (Li et al. 2021). In our study of multiple targets, the amplitude difference may be affected by the relative motion direction between tumors, which was not clearly observed in this study (Fig. 1) and further deepened the uncertainties. We investigated the correlation between intertarget position variation and both the unsynchronized tumor motion (intertarget amplitude difference) and the breathing amplitude variability (interfractional changes in amplitude difference). While a weak correlation with breathing amplitude variability was observed, the overall association was not obvious. This suggests that factors beyond geometric variations caused by respiratory motion may be at play. For time-resolved 4D images, breathing frequency may be a potential factor. Vergalasova et al. (2011) believed that the changes in inspiration to expiration ratio may lead to tumor localization errors during image acquisition. On the basis of all discussed above, to minimize intertarget uncertainties maintaining breathing stability and reproducibility over the treatment course is crucial, which means respiratory managements methods (e.g., gating, breath hold, abdominal compression, etc.) are also applicable to single-isocenter multitarget SBRT.

The location of tumor pairs also contributes to intertarget position variation. In consistent with the study by Van Timmeren et al. (2021), tumor pairs in the upper lobe exhibited less variation compared to subgroups with at least one tumor in the lower lobe. This aligns with established knowledge about tumor motion in different lung regions (Bissonnette et al. 2009), indicating that lower lobe tumor are more likely to be affected by the significant diaphragm motion Interestingly, the variations in the left lung was significantly higher compared to the right lung, even surpassing those in bilateral lung. The substantial variations observed in bilateral cases might be attributed to the increased inter-target distance. For left lung tumor pairs, the unexpected higher variations and risks compared to the right lung can be traced by the bronchial tree schema projected by Sonke et al. (2008). Their study showed that tumor motion in the left upper lobe can range from 10–15 mm, exceeding the right side by 5 mm. Geographically, the upper left lung is also more susceptible to the influence of heart contractions. Schmidt et al. (2016) observed that cardiac motion can introduce uncertainties predominant in AP direction. Reflecting on safety margin calculations, these larger systematic and random variations in the left lung necessitates the implementation of largest margin. To be noticed, we observed that a small number of patients exhibited significant intertarget position variations exceeding 10 mm during treatment fractions, as assessed by both 4D-CT (5.5%) and 4D-CBCT (3.4%). While the 4D-CT group exhibited a distinct lack of regularity in the distribution of these extreme values, within the 4D-CBCT group, patients with fewer tumors (*n* ≤ 3) were more likely to experience extreme variations. Four out of five patients with such variations had 2 or 3 tumors in either the upper and lower lobe of the left lung, or bilaterally. Only one patient with 5 lower lobe tumors in the right lung exhibited a single instance of extreme variation, likely an outlier. This suggests two possibilities: first, an increased number of tumors might limit the ability to correct their positions during image registration, though the direct link to variation is unclear. Second, and more likely, the location of the tumor pairs seems to be the key factor. Similarly, for the 5–10 mm variation range, left lung (32.3%) and bilateral (29.2%) tumor pairs share the highest risks of geometric miss, followed by the upper-lower lobe ( 26.9%) tumor pairs and the lower lobe (22.5%) tumor pairs. In conclusion, special attention should be given to tumor pairs in the left lung, as well as those in the lower lobes and bilateral lungs.

Due to the limitations of XVI version 5.0.3, the coordinates corrected 10-phase 4D-CBCT images cannot be transmitted to the planning system. We analyzed only the average tumor position variation which may underestimate the actual variation and the risk of geometric misses (Callahan et al. 2014). Considering that free-breathing CBCT images (3D-CBCT) are the most commonly used image guidance mode today, our results still provide rich information on practical treatment. In addition, the margins calculated in this study were based on limited samples, and future studies are mandatory before these parameters can be applied in clinical practice.

## Conclusions

This study provide a reliable way to assess intertarget position variation by using both 4D-CT and 4D-CBCT simulation. Consequently, tumor motion and RPEs constitute a substantial portion of intertarget position variation. 4D-CT simulation-based single-isocenter multitarget SBRT treatments without large margins should be applied cautiously. This quantitative assessment informs future strategies for minimizing geometric miss rates in single-isocenter multi-target SBRT.

## Supplementary Information

Below is the link to the electronic supplementary material.

Supplementary file1 (JPG 658 KB)

Supplementary file2 (JPG 142 KB)

## Data Availability

All the datasets are available from the corresponding author on reasonable request.
