# Supplementary figures and images for: Quantitative assessment of intertarget position variations based on 4D-CT and 4D-CBCT simulations in single-isocenter multitarget lung stereotactic body radiation therapy
Source: J Cancer Res Clin Oncol. 2024 Jul 24;150(7):359. doi: 10.1007/s00432-024-05836-w (PMC11266286; doi:10.1007/s00432-024-05836-w)

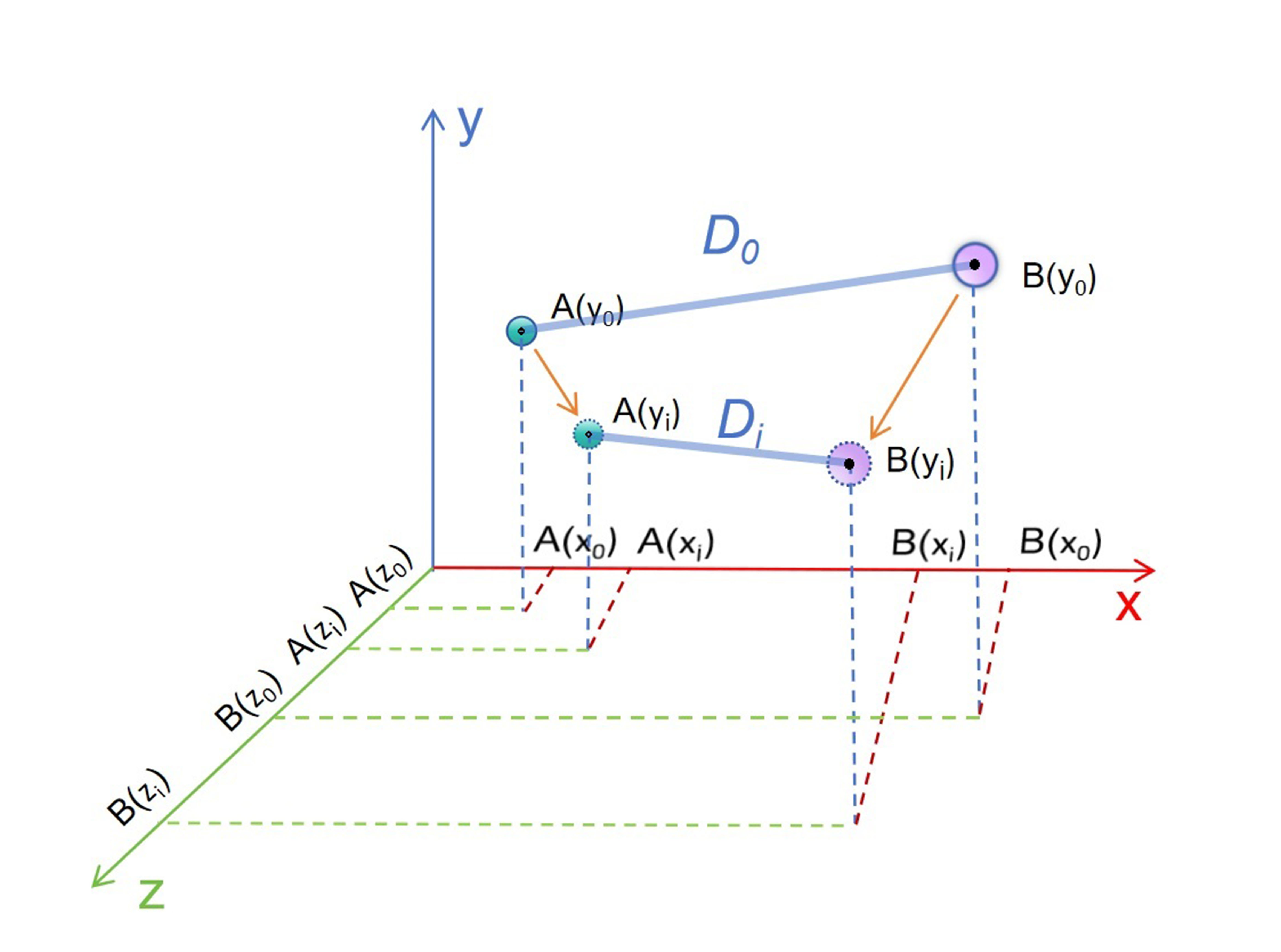

Supplement: Supplementary file 1 — Supplementary file1 (JPG 658 KB) [file 432_2024_5836_MOESM1_ESM.jpg]

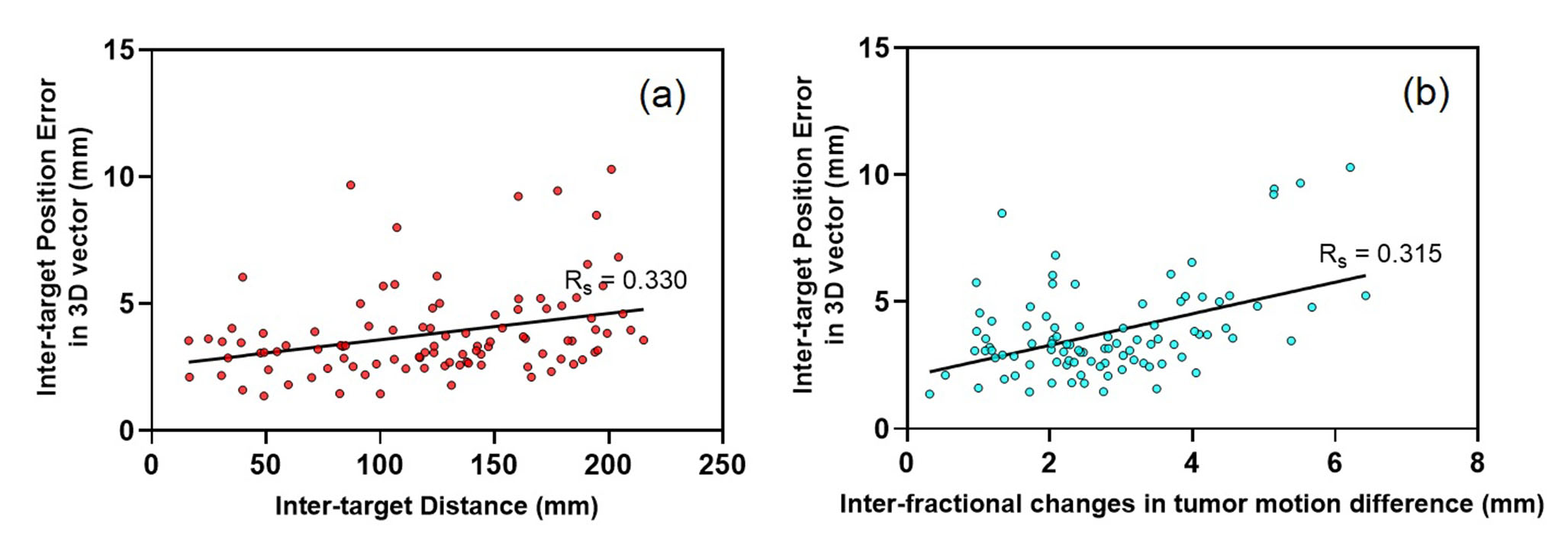

Supplement: Supplementary file 2 — Supplementary file2 (JPG 142 KB) [file 432_2024_5836_MOESM2_ESM.jpg]
